# *BRIP1* coding variants are associated with a high risk of hepatocellular carcinoma occurrence in patients with HCV- or HBV-related liver disease

**DOI:** 10.18632/oncotarget.11327

**Published:** 2016-08-17

**Authors:** Abderrahim Oussalah, Patrice Hodonou Avogbe, Erwan Guyot, Céline Chery, Rosa-Maria Guéant-Rodriguez, Nathalie Ganne-Carrié, Aurélie Cobat, Darius Moradpour, Bertrand Nalpas, Francesco Negro, Thierry Poynard, Stanislas Pol, Pierre-Yves Bochud, Laurent Abel, Hélène Jeulin, Evelyne Schvoerer, Nicodème Chabi, Emile Amouzou, Ambaliou Sanni, Hélène Barraud, Pierre Rouyer, Thomas Josse, Laetitia Goffinet, Jean-Louis Jouve, Anne Minello, Claire Bonithon-Kopp, Gérard Thiefin, Vincent Di Martino, Michel Doffoël, Carine Richou, Jean-Jacques Raab, Patrick Hillon, Jean-Pierre Bronowicki, Jean-Louis Guéant, for the CiRCE Study Group

**Affiliations:** ^1^ INSERM, U954, NGERE – Nutrition, Genetics, and Environmental Risk Exposure, Faculty of Medicine of Nancy, University of Lorraine, Vandoeuvre-lès-Nancy, France; ^2^ Department of Molecular Medicine and Personalized Therapeutics, Department of Biochemistry, Molecular Biology, Nutrition, and Metabolism, University Hospital of Nancy, Vandoeuvre-lès-Nancy, France; ^3^ Biochemistry Unit, Jean Verdier Hospital, APHP, Bondy, France and University Paris 13-UFR SMBH/INSERM, Bobigny, France; ^4^ Liver Unit and Liver biobank CRB des Hôpitaux Universitaires Paris-Seine-Saint-Denis BB-0033-00027, Jean Verdier Hospital, APHP, Bondy, France; ^5^ INSERM, U1162, Génomique fonctionnelle des Tumeurs solides, Paris, France; ^6^ Laboratory of Human Genetics of Infectious Diseases, Necker Branch, INSERM U1163, Paris, France; ^7^ Paris Descartes University, Imagine Institute, Paris, France; ^8^ Division of Gastroenterology and Hepatology, University Hospital and University of Lausanne, Switzerland; ^9^ Département d'Hépatologie, Hôpital Cochin (AP-HP), Université Paris Descartes, Paris, France; ^10^ Division of Clinical Pathology and Division of Gastroenterology and Hepatology, University Hospitals, Geneva, Switzerland; ^11^ Université Pierre et Marie Curie, Service d'Hépato-gastroentérologie, Hôpital Pitié-Salpêtrière (AP-HP), Paris, France; ^12^ INSERM UMS20, Institut Pasteur, Paris, France; ^13^ Infectious Diseases Service, Department of Medicine, University Hospital and University of Lausanne, Switzerland; ^14^ St. Giles Laboratory of Human Genetics of Infectious Diseases, Rockefeller Branch, The Rockefeller University, NY, USA; ^15^ Virology Laboratory, Centre Hospitalier Universitaire de Nancy, Vandoeuvre-lès-Nancy, France; ^16^ Laboratory of Biochemistry and Molecular Biology, University of Cotonou, Cotonou, Benin; ^17^ Laboratory of Biochemistry and Nutrition, Lomé, University of Kara, Togo; ^18^ Department of Hepato-Gastroenterology, University Hospital of Nancy, Vandoeuvre-lès-Nancy, France; ^19^ INSERM, U866 and INSERM, CIE 01, University Hospital of Dijon, University of Burgundy, Dijon, France; ^20^ Department of Hepato-Gastroenterology, Reims University Hospital, Reims, France; ^21^ Department of Hepatology, University Hospital of Besançon, Besançon, France; ^22^ Department of Hepato-Gastroenterology, University Hospital of Strasbourg, Strasbourg, France; ^23^ Regional Hospital of Metz, Metz, France

**Keywords:** DNA repair genes, hepatocellular carcinoma, BRIP1, hepatitis B virus, hepatitis C virus

## Abstract

The molecular mechanisms of hepatocellular carcinoma (HCC) carcinogenesis are still not fully understood. DNA repair defects may influence HCC risk. The aim of the study was to look for potential genetic variants of DNA repair genes associated with HCC risk among patients with alcohol- or viral-induced liver disease. We performed four case-control studies on 2,006 European- (Derivation#1 and #2 studies) and African-ancestry (Validation#1 and #2 studies) patients originating from several cohorts in order to assess the association between genetic variants on DNA repair genes and HCC risk using a custom array encompassing 94 genes. In the Derivation#1 study, the *BRIP1* locus reached array-wide significance (Chi-squared SV-Perm, *P*=5.00×10^−4^) among the 253 haplotype blocks tested for their association with HCC risk, in patients with viral cirrhosis but not among those with alcoholic cirrhosis. The *BRIP1* haplotype block included three exonic variants (rs4986763, rs4986764, rs4986765). The *BRIP1* ‘*AAA*’ haplotype was significantly associated with an increased HCC risk [odds ratio (OR), 2.01 (1.19–3.39); false discovery rate (FDR)-*P*=1.31×10^−2^]. In the Derivation#2 study, results were confirmed for the *BRIP1* ‘*GGG*’ haplotype [OR, 0.53 (0.36–0.79); FDR-*P*=3.90×10^−3^]. In both Validation#1 and #2 studies, *BRIP1* ‘*AAA*’ haplotype was significantly associated with an increased risk of HCC [OR, 1.71 (1.09–2.68); FDR-*P*=7.30×10^−2^; and OR, 6.45 (4.17–9.99); FDR-*P*=2.33×10^−19^, respectively]. Association between the *BRIP1* locus and HCC risk suggests that impaired DNA mismatch repair might play a role in liver carcinogenesis, among patients with HCV- or HBV-related liver disease.

## INTRODUCTION

Hepatocellular carcinoma (HCC) is the commonest primary malignant tumor of the liver. It is the fifth most common cancer in men and the seventh in women, and it ranks third in annual cancer mortality rates worldwide [1, 2]. Infection with hepatitis C virus (HCV) or hepatitis B virus (HBV), alcoholic liver disease, and nonalcoholic fatty liver disease are the most dominant risk factors for HCC development [2]. The highest incidence rates of HCC occur in developing countries, such as sub-Saharan Africa, where the prevalence of chronic viral hepatitis is high [3]. The molecular mechanisms of HCC carcinogenesis are still not fully understood and are different according to their origins [4, 5]. HCC risk factors induce malignant transformation of hepatocytes by increasing cellular turnover leading to multiple genetic alterations such as chromosomal instability with point mutations and deletions causing the activation or inactivation of proto-oncogenes or tumor suppressor genes, respectively [5]. HCV- and HBV-related HCC develop in an environment of ongoing inflammation and cell injury, both leading to increased cell turnover and liver fibrosis [6]. It has been shown that HCV can induce alteration of genes involved in deoxyribonucleic acid (DNA) mismatch repair (MMR) and cell cycle regulation [5, 7, 8]. The pathogenesis of HCC associated with alcoholic cirrhosis follows other mechanistic pathways that mainly involve oxidative stress in relation with ethanol metabolism and inflammation [9].

It has been suggested that genetic variants related to DNA repair could modulate HCC risk [10]. DNA repair systems are important parts of the cellular defense against a large variety of structural unrelated DNA lesions generated by both exogenous (ionizing radiation, strong alkylating agents) and endogenous DNA-damaging agents (viruses) [4, 11]. Genetic polymorphisms in DNA repair genes may influence individual variation in DNA repair capacity and may play an important role in carcinogenesis [12]. Several case-control studies evaluated the association between polymorphisms in DNA repair genes and HCC risk and have been inconclusive [13–19]. However, these studies have been limited to a single or a small number of genes and focused only on three DNA repair gene pathways, namely, base excision repair (BER), nucleotide excision repair (NER), and double-strand breaks repair (DSBR) [13–19].

Both candidate-gene and genome-wide association approaches have been used to identify common genetic variants associated with HCC risk [13–22]. Three genome-wide association studies (GWASs) conducted in Asian populations looked for genetic variants potentially associated with HCC in patient with HBV- or HCV-induced chronic viral hepatitis [20–22]. These studies have failed to clarify the pathogenic pathways associated with HCC. One study was negative [21], a second study found intergenic variants outside any pathogenic pathway [22] and the third reported an association with a genetic variant on a gene involved in neurotransmission [20]. Furthermore, no GWAS has been performed in European- or in African-ancestry populations.

We hypothesized that the inconclusive studies on the association between genetic variants in DNA repair genes and HCC risk may reflect limitations in study design and genetic variant selection. Using data from cirrhotic patients with and without HCC and non-cirrhotic patients with HCV- and/or HBV-related disease, we assessed the association between genetic variants on DNA repair genes and HCC risk using an in-house designed array. Through the same study design, we followed the derivation studies on European-ancestry patients by successful validation studies on African-ancestry patients that confirmed the strong implication of the *BRIP1* locus in the genetic determinism of HCC risk in patients with HCV- and/or HBV-related liver disease.

## RESULTS

### Derivation #1 study among patients with alcoholic cirrhosis

On the 496 cirrhotic patients included in the study (Table 1), 355 had alcoholic cirrhosis (See Supplemental Table S1 in the supplementary appendix for patients’ characteristics). Among patients with alcoholic cirrhosis, 112 had HCC and were compared with 243 HCC-free patients. No haplotype block reached array-wide significance among the 253 haplotype blocks analyzed.

**Table 1:** Characteristics of cirrhotic patients with and without hepatocellular carcinoma in the Derivation #1 study

	Whole population(n=496)		Cirrhotic patientswith HCC (n=168)		Cirrhotic patientswithout HCC (n=328)		
	Median	IQR	Median	IQR	Median	IQR	P-value*
Year of cirrhosis diagnosis	2008	2005 to 2009	2008	2005 to 2009	2007	2005 to 2009	—
Age, years	60	54 to 68	65	59 to 72	58	52 to 65	<0.0001
Weight, kg	78	66 to 90	80	67 to 92	76	66 to 89	0.17
Height, m	1.70	1.65 to 1.76	1.71	1.65 to 1.77	1.70	1.65 to 1.76	0.62
BMI (kg/m^2^)	26.6	23.5 to 29.9	27.6	23.4 to 30.7	26.2	23.6 to 29.7	0.13
	n/N (%)	95% CI	n/N (%)	95% CI	n/N (%)	95% CI	P-value^†^
***Gender***							
Female	128/496 (25.8)	21.9 to 29.7	29/168 (17.3)	11.5 to 23.0	99/328 (30.2)	25.2 to 35.2	0.002
Male	368/496 (74.2)	70.3 to 78.1	139/168 (82.7)	77 to 88.5	229/328 (69.8)	64.8 to 74.8	—
***Alcohol consumption***							
Yes	456/494 (92.3)	89.9 to 94.7	156/168 (92.9)	88.9 to 96.8	300/326 (92.0)	89.1 to 95	0.86
No	38/494 (7.7)	5.3 to 10.1	12/168 (7.1)	3.2 to 11.1	26/326 (8.0)	5 to 10.9	—
***Smoking status***							
Current	189/494 (38.3)	34 to 42.6	54/167 (32.3)	25.2 to 39.5	135/327 (41.3)	35.9 to 46.6	0.06
Former	179/494 (36.2)	32 to 40.5	77/167 (46.1)	38.5 to 53.7	102/327 (31.2)	26.1 to 36.2	0.002
Never	126/494 (25.5)	21.6 to 29.4	36/167 (21.6)	15.3 to 27.9	90/327 (27.5)	22.7 to 32.4	0.16
***Etiology of cirrhosis***							
Alcohol	355/496 (71.6)	67.6 to 75.6	112/168 (66.7)	59.5 to 73.9	243/328 (74.1)	69.3 to 78.9	‡
HCV	115/496 (23.2)	19.5 to 26.9	42/168 (25.0)	18.4 to 31.6	73/328 (22.3)	17.7 to 26.8	‡
HBV	23/496 (4.6)	2.8 to 6.5	13/168 (7.7)	3.7 to 11.8	10/328 (3.1)	1.2 to 4.9	‡
HCV and HBV coinfection	3/496 (0.6)	0 to 1.3	1/168 (0.6)	0 to 1.8	2/328 (0.6)	0 to 1.46	‡

**NOTE. BMI**: Body mass index; **HCC**: hepatocellular carcinoma; **HCV**: hepatitis C virus; **HBV**; hepatitis B virus; **IQR**: interquartile range.

* Mann-Whitney *U* test; † Chi-squared test; ‡ Proportions of cirrhosis etiologies have not been compared between cases and controls because patients were selected on the basis of this criterion.

### Derivation #1 study among patients with viral cirrhosis

Characteristics of patients with viral cirrhosis according to their HCC status are reported in Table 2. Patients with HCV-related cirrhosis did not differ from those with HBV-related cirrhosis regarding baseline characteristics (See Supplemental Table S2 in the supplementary appendix).

**Table 2:** Characteristics of patients with viral cirrhosis according to their hepatocellular carcinoma status in the Derivation #1 study

	Whole population(n=141)		Cirrhotic patientswith HCC (n=56)		Cirrhotic patientswithout HCC (n=85)		
	**Median**	**IQR**	**Median**	**IQR**	**Median**	**IQR**	*P*-value*
Year of cirrhosis diagnosis	2007	2003 to 2009	2007	2003 to 2009	2007	2003 to 2008	0.34
Age, years	62	53 to 71	67	55 to 74	61	51 to 68	0.03
Weight, kg	74	65 to 84	71	62 to 84	75	67 to 85	0.11
Height, m	1.70	162 to 175	1.69	161 to 175	1.70	162 to 176	0.81
BMI (kg/m^2^)	25.3	23.0 to 29.1	24.4	21.8 to 28.7	26.2	23.8 to 29.5	0.07
ALAT (IU/L)	55	32 to 94	57	30 to 94	54	33. to 97	0.95
Disease duration (years)	2	1 to 7	2	0 to 7	2.000	1 to 7	0.17
	n/N (%)	95% CI	n/N (%)	95% CI	n/N (%)	95% CI	*P*-value†
***Gender***							
Female	39/141 (27.7)	20.2 to 35.1	17/56 (30.4)	17.9 to 42.8	22/85 (25.9)	16.4 to 35.4	0.70
Male	102/141 (72.3)	64.9 to 79.8	39/56 (69.6)	57.2 to 82.1	63/85 (74.1)	64.6 to 83.6	—
***Etiology of cirrhosis***							
HCV	115/141 (81.6)	75.1 to 88	42/56 (75.0)	63.3 to 86.7	73/85 (85.9)	78.3 to 93.4	0.16
HBV	23/141 (16.3)	10.1 to 22.5	13/56 (23.2)	11.8 to 34.6	10/85 (11.8)	4.77 to 18.8	0.12
HCV and HBV coinfection	3/141 (2.13)	0 to 4.54	1/56 (1.79)	0 to 5.36	2/85 (2.35)	0 to 5.64	‡
***Alcohol consumption***							
Yes	110/140 (78.6)	71.7 to 85.5	44/56 (78.6)	67.5 to 89.7	66/84 (78.6)	69.6 to 87.5	0.83
No	30/140 (21.4)	14.5 to 28.3	12/56 (21.4)	10.3 to 32.5	18/84 (21.4)	12.5 to 30.4	—
***Smoking status***							
Current	51/141 (36.2)	28.1 to 44.2	22/56 (39.3)	26.1 to 52.5	29/85 (34.1)	23.8 to 44.4	0.66
Former	50/141 (35.5)	27.5 to 43.5	15/56 (26.8)	14.8 to 38.8	35/85 (41.2)	30.5 to 51.9	0.12
Never	40/141 (28.4)	20.8 to 35.9	19/56 (33.9)	21.1 to 46.7	21/85 (24.7)	15.3 to 34.1	0.32

**NOTE. HCC**: hepatocellular carcinoma; **ALAT**: alanine aminotransferase; **BMI**: Body mass index; **HCV**: hepatitis C virus; HBV; hepatitis B virus; IQR: interquartile range.

* Mann-Whitney *U* test; † Chi-squared test; ‡ Statistical comparison not performed due low sample size.

**Figure 1:**
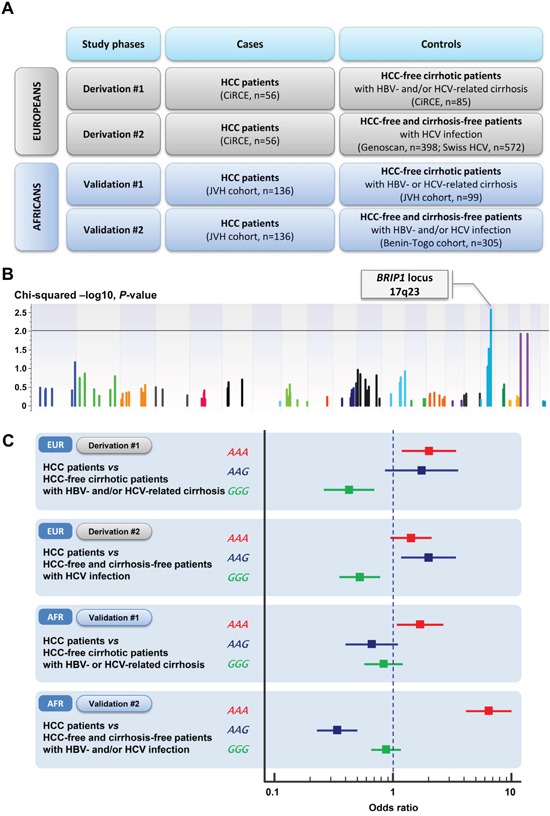
**A**. Overview on the study design; **B**. Manhattan plot reporting the association between haplotype blocks on DNA repair genes and hepatocellular carcinoma risk in the Derivation #1 study among patients with viral cirrhosis. Association results of the single-variant analysis (−Log10 P) are plotted against genomic position (GRCh37 hg19); **C**. Forest plots showing the effect size of associations between HHC risk and *BRIP1* haplotypes in derivation (Derivation #1 and #2) and validation (Validations #1 and #2) studies among patients with viral hepatitis. For each analysis, the odds ratio of the association is reported with the 95% confidence interval (solid line) [**EUR**: Europeans; **AFR**: Africans; **HCC**: hepatocellular carcinoma; **HBV**: hepatitis B virus; **HCV**: hepatitis C virus; **Derivation #1**: HCC patients from the CiRCE study (n=56) were compared with HBV- and/or HCV-induced cirrhosis but without HCC from the CiRCE study (n=85); **Derivation #2**: HCC patients from the CiRCE study (n=56) were compared with patients with HCV-related chronic liver disease from two European cohorts (ANRS Genoscan study group, n=398; Swiss Hepatitis C Cohort Study, n=572); **Validation #1**: HCC patients from the Jean Verdier Hospital cohort (n=136) were compared with HCC-free cirrhotic patients (Jean Verdier Hospital cohort) (n=99); **Validation #2**: HCC patients from the Jean Verdier Hospital cohort (n=136) were compared with HBV- and/or HCV-infection from the Benin-Togo cohort (n=305)].

Among patients with viral cirrhosis, 56 HCC patients were compared with 85 HCC-free patients. Among the 253 haplotype blocks tested for their association with HCC risk only one reached array-wide significance and was located in the *BRIP1* locus (BRCA1 interacting protein C-terminal helicase 1; Gene ID: 83990; formerly known as *BACH1* or *FANCJ*) in the chromosome 17q23 (Chi-Squared SV Perm, *P*=5.00×10^−4^) (See Supplemental Table S3 in the supplementary appendix). The *BRIP1* haplotype block included three exonic variants with two variants located in the exon 19 (rs4986765, p.Glu879= and rs4986764, p.Ser919Pro) and one variant located in the exon 20 (rs4986763; p.Tyr1137=). These three variants were in strong LD within the *BRIP1* haplotype block with D’ values ranging from 0.97 to 1. Among the three different haplotypes of the *BRIP1* haplotype block, the ‘*AAA*’ haplotype (simultaneous occurrence of the minor allele for each one of the three *BRIP1* variants) was associated with an increased risk of HCC (EM frequency in cases, 38%; EM frequency in controls, 24%; odds ratio, 2.01; 95% CI, 1.19 to 3.39; *P*=8.74×10^−3^; FDR-*P*=1.31×10^−2^). Conversely, the *BRIP1* ‘*GGG*’ haplotype (simultaneous occurrence of the major allele for each one of the three *BRIP1* variants) was associated with a decreased HCC risk (EM frequency in cases, 45%; EM frequency in controls, 66%; odds ratio, 0.42; 95% CI, 0.26 to 0.69; *P*=5.09×10^−4^; FDR-*P*=1.53×10^−3^) (Table 3, Figure 1, and Supplemental Figure S1 in the supplementary appendix).

**Table 3:** Association between *BRIP1* haplotypes and HCC risk in derivation (Derivation #1 and #2) and validation (Validation #1 and #2) studies among patients with viral hepatitis

*BRIP1*Haplotype*	EMfrequency,Cases	EMfrequency,Controls	Chi-Squared*P*-value	Chi-SquaredFDR	Oddsratio^†^	95% CI,Odds ratio
**Derivation #1 study (EUR)**^‡^**HCC patients vs HCC-free cirrhotic patients with HBV- and/or HCV-related cirrhosisPer block, *P*-value = 5.00×10^−4^**						
*AAA*	0.38	0.24	8.74×10^−3^	1.31×10^−2^	2.01	1.19 to 3.39
*AAG*	0.16	0.10	0.12	0.12	1.76	0.86 to 3.58
*GGG*	0.45	0.66	5.09×10^−4^	1.53×10^−3^	0.42	0.26 to 0.69
**Derivation #2 study (EUR)**^§^**HCC patients vs HCC-free and cirrhosis-free patients with HCV infectionPer block, *P*-value = 1.93×10^−3^**						
*AAA*	0.38	0.30	0.08	0.08	1.43	0.96 to 2.13
*AAG*	0.16	0.09	9.05×10^−3^	1.36×10^−2^	2.00	1.18 to 3.39
*GGG*	0.45	0.60	1.30×10^−3^	3.90×10^−3^	0.53	0.36 to 0.79
**Validation #1 study (AFR)**^‖^**HCC patients vs HCC-free cirrhotic patients with HBV- or HCV-related cirrhosisPer block, *P*-value = 0.08**						
*AAA*	0.28	0.18	1.82×10^−2^	7.30×10^−2^	1.71	1.09 to 2.68
*AAG*	0.13	0.18	0.12	0.23	0.66	0.40 to 1.11
*GGG*	0.58	0.63	0.35	0.47	0.84	0.57 to 1.22
**Validation #2 study (AFR)^¶^HCC patients vs HCC-free and cirrhosis-free patients with HBV- and/or HCV infectionPer block, *P*-value = 4.47×10^−21^**						
*AAA*	0.28	0.06	5.82×10^−20^	2.33×10^−19^	6.45	4.17 to 9.99
*AAG*	0.13	0.30	3.81×10^−8^	7.63×10^−8^	0.34	0.23 to 0.50
*GGG*	0.58	0.62	0.33	0.33	0.86	0.65 to 1.16

**NOTE. HCC**: hepatocellular carcinoma; **EM**: haplotype frequency using the expectation/maximization algorithm; **FDR**: false discovery rate; **95% CI**: 95% confidence interval; **EUR:** Europeans, **AFR:** Africans; **JVH:** Jean Verdier Hospital cohort.

* *BRIP1* haplotypes are based on the three *BRIP1* variants studied sorted by increasing genomic position (rs4986763, rs4986764, rs4986765)

† Odds ratio for patients harboring the specified haplotype

‡ Derivation #1: HCC patients from the CiRCE study (n=56) were compared with HBV- and/or HCV-induced cirrhosis but without HCC from the CiRCE study (n=85)

§ Derivation #2: HCC patients from the CiRCE study (n=56) were compared with patients with HCV-related chronic liver disease from two European cohorts (ANRS Genoscan study group, n=398; Swiss Hepatitis C Cohort Study, n=572)

‖ Validation #1: HCC patients from the Jean Verdier Hospital cohort (n=136) were compared with HCC-free cirrhotic patients (Jean Verdier Hospital cohort) (n=99)

¶ Validation #2: HCC patients from the Jean Verdier Hospital cohort (n=136) were compared with HBV- and/or HCV-infection from the Benin-Togo cohort (n=305)

In the Derivation #1 study among patients with viral cirrhosis, harboring the minor allele for the *BRIP1* variant (rs4986764; p.Ser919Pro) conferred an increased risk of HCC (odds ratio, 2.32; 95% CI, 1.42 to 3.79; *P*=8.32×10^−4^) (Table 4).

**Table 4:** Association between *BRIP1* genetic variants and HCC risk in derivation (Derivation #1 and #2) and validation (Validation #1 and #2) studies among patients with viral hepatitis

Variant	Chr	Position*	Residue change	Fisher's Exact *P*-value	Fisher's Exact FDR	Oddsratio^†^	95% CI	MAF, Cases	MAF, Controls
**Derivation #1 study (EUR)**^‡^**HCC patients vs HCC-free cirrhotic patients with HBV- and/or HCV-related cirrhosis**									
rs4986763	17	59760996	p.Tyr1137=	4.96×10^−4^	1.49×10^−3^	2.42	1.48 to 3.97	0.55	0.34
rs4986764	17	59763347	p.Ser919Pro	8.32×10^−4^	1.25×10^−3^	2.32	1.42 to 3.79	0.55	0.34
rs4986765	17	59763465	p.Glu879=	1.06×10^−2^	1.06×10^−2^	2.01	1.19 to 3.39	0.38	0.24
**Derivation #2 study (EUR)**^§^**HCC patients vs HCC-free and cirrhosis-free patients with HCV infection**									
rs4986763	17	59760996	p.Tyr1137=	1.86×10^−3^	5.59×10^−3^	1.88	1.27 to 2.76	0.55	0.40
rs4986764	17	59763347	p.Ser919Pro	2.59×10^−3^	3.89×10^−3^	1.83	1.24 to 2.69	0.55	0.40
rs4986765	17	59763465	p.Glu879=	0.09	0.09	1.41	0.95 to 2.10	0.38	0.30
**Validation #1 study (AFR)**^‖^**HCC patients vs HCC-free cirrhotic patients with HBV- or HCV-related cirrhosis**									
rs4986763	17	59760996	p.Tyr1137=	0.45	0.45	1.17	0.81 to 1.71	0.41	0.37
rs4986764	17	59763347	p.Ser919Pro	0.34	0.51	1.21	0.83 to 1.77	0.40	0.36
rs4986765	17	59763465	p.Glu879=	2.19×10^−2^	6.56×10^−2^	1.69	1.08 to 2.63	0.28	0.19
**Validation #2 study (AFR) ^¶^HCC patients vs HCC-free and cirrhosis-free patients with HBV- and/or HCV infection**									
rs4986763	17	59760996	p.Tyr1137=	0.33	0.33	1.16	0.86 to 1.55	0.41	0.38
rs4986764	17	59763347	p.Ser919Pro	0.17	0.26	1.23	0.92 to 1.65	0.40	0.36
rs4986765	17	59763465	p.Glu879=	6.42×10^−18^	1.93×10^−17^	6.18	4.03 to 9.49	0.28	0.06

**NOTE. Chr**: chromosome; **FDR**: false discovery rate; **OR**: odds ratio; **95% CI**: 95% confidence interval; **MAF**: minor allele frequency.

* According to the GRCh37 assembly

† Odds ratio for the minor allele

‡ Derivation #1: HCC patients from the CiRCE study (n=56) were compared with HBV- and/or HCV-induced cirrhosis but without HCC from the CiRCE study (n=85)

§ Derivation #2: HCC patients from the CiRCE study (n=56) were compared with patients with HCV-related chronic liver disease from two European cohorts (ANRS Genoscan study group, n=398; Swiss Hepatitis C Cohort Study, n=572)

‖ Validation #1: HCC patients from the Jean Verdier Hospital cohort (n=136) were compared with HCC-free cirrhotic patients (Jean Verdier Hospital cohort) (n=99)

¶ Validation #2: HCC patients from the Jean Verdier Hospital cohort (n=136) were compared with HBV- and/or HCV-infection from the Benin-Togo cohort (n=305)

In multivariate logistic regression analysis, the three *BRIP1* variants were independently associated with HCC risk after adjusting for age, sex, body mass index, alcohol consumption, alanine aminotransferase level, disease duration of cirrhosis, viral cirrhosis etiology, and the top eigenvalue obtained from the primary component analysis in order to adjust for potential population admixture (rs4986763, adjusted OR, 2.52; 95% CI, 1.37 to 4.61; *P*=2.9×10^−3^; rs4986764, adjusted OR, 2.50; 95% CI, 1.36 to 4.59; *P*=3.2×10^−3^; and rs4986765, adjusted OR, 2.18; 95% CI, 1.17 to 4.06; *P*=1.4×10^−2^).

**Figure 2:**
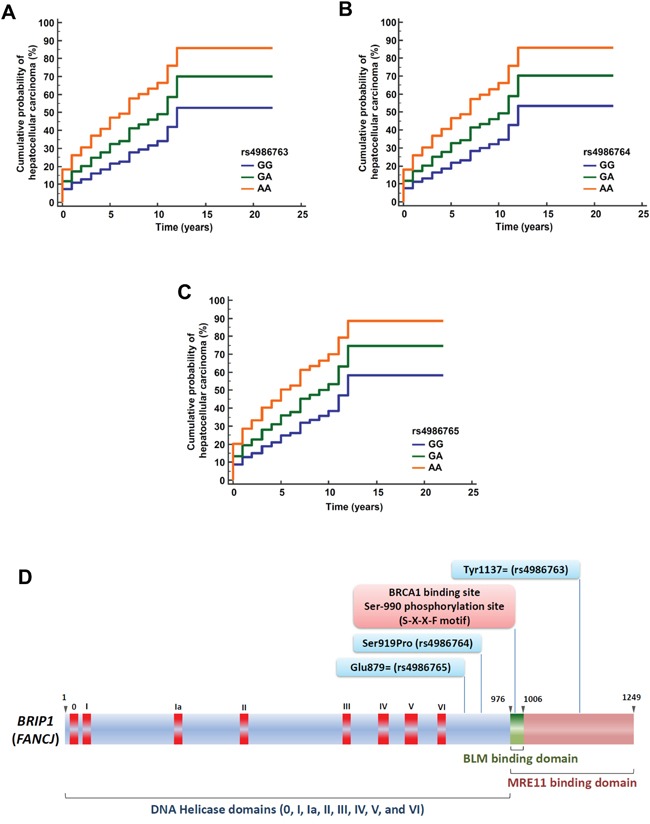
Cumulative probability of hepatocellular carcinoma occurrence among patients with viral cirrhosis in the CiRCE study according to BRIP1 variants Cumulative probability of hepatocellular carcinoma occurrence according to *BRIP1* variants [rs4986763 **(A)**, rs4986764 **(B)**, and rs4986765 **(C)**] among patients with viral cirrhosis using multivariate Cox proportional-hazards regression analysis; *GG*: Major homozygous genotype, *GA*: Heterozygous genotype, *AA*: Minor homozygous genotype; (**D**) Structure of the *BRIP1* gene. The *BRIP1* gene codes for the BRIP1 protein which comprises DNA Helicase domains (0, I, Ia, II, III, IV, V, and VI), a BLM binding domain, and MRE11 binding domain. Genomic positions of the three *BRIP1* variants found in association with hepatocellular cancer risk among patients with viral cirrhosis are indicated (adapted from references [24, 27, 58]).

Using multivariate Cox proportional-hazards regression analysis, the three *BRIP1* variants were independently associated with time to HCC occurrence after adjusting for age, sex, body mass index, alcohol consumption, aminotransferase level, viral cirrhosis etiology, and the top eigenvalue obtained from the primary component analysis in order to adjust for potential population admixture (rs4986763, adjusted HR, 1.62; 95% CI, 1.06 to 2.47; *P*=2.6×10^−2^; rs4986764, adjusted HR, 1.60; 95% CI, 1.05 to 2.44; *P*=3.2×10^−2^; rs4986765, adjusted HR, 1.57; 95% CI, 1.03 to 2.42; *P*=3.8×10^−2^) (Figure 2).

### Derivation #2 study

In the Derivation #2 study, *BRIP1* haplotypes ‘*AAA*’ and ‘*GGG*’ had similar effect sizes for their association with HCC risk in comparison with those observed in Derivation #1 study (Table 3). Consistently, at the genetic variant level, harboring the minor allele for the *BRIP1* variant (rs4986764; p.Ser919Pro) conferred an increased risk of HCC (odds ratio, 1.83; 95% CI, 1.24 to 2.69; *P*=2.59×10^−3^) (Table 4). In post-hoc exploratory analysis, haplotype trend regression was carried out in order to estimate the influence of HCV genotype on the association between *BRIP1* haplotypes and HCC phenotype in a subset of patients with available data from the Derivation#2 study (23 cases from CiRCE and 970 controls from the ANRS Genoscan and Swiss Hepatitis C Cohort Studies). Consistently, the *BRIP1* ‘*GGG’* haplotype was still significantly associated with a reduced risk of HCC risk (odds ratio, 0.18; 95% CI, 0.05 to 0.59; *P*=4.16×10^−3^). HCV genotype did not influence this association (*P*=0.26).

### Validation #1 study

In the Validation #1 study on African ancestry patients, 136 HCC patients with viral-related cirrhosis from the JVH cohort were compared with 99 HCC-free patients with HBV- or HCV-related cirrhosis from the JVH cohort. Among cirrhotic patients, the proportions of HCV- and HBV-related cirrhosis were 57.4% (n=135) and 42.6% (n=100), respectively and males were predominant (65.1%, n=153). The *BRIP1* haplotype block was significantly associated with HCC risk. The *BRIP1* ‘*AAA*’ haplotype was associated with an increased risk of HCC (EM frequency in cases, 28%; EM frequency in controls, 18%; odds ratio, 1.71; 95% CI, 1.09 to 2.68; *P*=1.82×10^−2^; FDR-*P*=7.30×10^−2^) (Table 3). At the genetic variant level, on the three *BRIP1* variants tested for their association with HCC risk, only rs4986765 was significantly associated with HCC risk (odds ratio, 1.69; 95% CI, 1.08 to 2.63; *P*=2.19×10^−2^; FDR-*P*=6.56×10^−2^) (Table 4).

### Validation #2 study

In the Validation #2 study on African ancestry patients, 136 HCC patients with viral-related cirrhosis from the JVH cohort were compared with 305 HCC-free and cirrhosis-free patients with HBV- and/or HCV infection recruited in Benin-Togo cohort. Consistently with results from the Validation #1 study, the *BRIP1* ‘*AAA*’ haplotype was associated with an increased risk of HCC (EM frequency in cases, 28%; EM frequency in controls, 6%; odds ratio, 6.45; 95% CI, 4.17 to 9.99; *P*=5.82×10^−20^; FDR-*P*=2.33×10^−19^) (Table 3). At the genetic variant level, on the three *BRIP1* variants tested for their association with HCC risk, only rs4986765 was significantly associated with HCC risk (odds ratio, 6.18; 95% CI, 4.03 to 9.49; *P*=6.42×10^−18^; FDR-*P*=1.93×10^−17^) (Table 4).

## DISCUSSION

This is the first study that has performed a comprehensive assessment of DNA repair genes variants and their influence on HCC risk in both European- and African-ancestry populations. We demonstrated that the *BRIP1* locus was strongly associated with HCC risk in patients with HBV- and/or HCV-induced liver disease even after adjusting for age, sex, body mass index, alcohol consumption, aminotransferase level, disease duration, viral cirrhosis etiology, and potential population admixture. *BRIP1*, also known as *BACH1* or *FANCJ*, is an essential tumor suppressor gene based on the identification of clinically relevant *BRIP1* mutations in several cancers like hereditary breast and ovarian cancers and childhood cancer syndrome [23]. *BRIP1* encodes a 1249 residue nuclear protein (BRIP1) which collaborates with a number of DNA metabolizing proteins implicated in DNA damage detection and repair, and plays an important role in cell cycle checkpoint control [24]. BRIP1 is a DNA-dependent ATPase and a 5′-to-3′ DNA helicase that achieves its cancer suppression function and DNA double-strand break repair through direct interaction with the highly conserved, C-terminal BRCT (BRCA1 C-Terminal domain) protein domain repeats of the tumor suppressor BRCA1 [24, 25]. The N-terminal 888-residue structure of BRIP1 shows strong homology with the catalytic and nucleotide binding domain of DEAH helicase family members [26, 27]. Abnormal BRIP1 function contributes to tumor induction [25]. Indeed, mutations within the BRCT repeats on BRCA1 disrupt its interaction with BRIP1 and lead to defects in DNA repair thereby resulting in several forms of cancers [25]. In two patients with early-onset breast cancer, mutations in the *BRIP1* coding regions resulted in a defective helicase activity [25]. Hence, both absence of the BRIP1 DNA helicase function or BRIP1-BRCA1 interaction can induce defects in several aspects of the DNA damage response [23].

Phosphorylation of the BRIP1 protein mediates interactions that promote repair and check point responses [24]. Phosphorylation of the Serine residue at position 990 is essential for BRIP1 binding to the tandem C-terminal BRCT motifs of BRCA1 [28] (Figure 2D). Loss of the Ser-990 phosphorylation limits homologous recombination repair of double strand break [24, 29]. The BRCA1-interacting region of BRIP1 extends from residues 976 to 1006 that corresponds to exons 19 and 20 (Figure 2D) [24]. In our study, the three *BRIP1* variants that we found in association with HCC risk are proxy to the BLM (Bloom's syndrome protein) domain that overlaps with the BRIP1 Ser-990 phosphorylation site, raising the hypothesis that the protein interaction of BRIP1 with BLM is affected by phosphoSer-990 [24, 30].

In our study, the *BRIP1* variant rs4986763 is located in the MRE11 protein binding domain. MRE11 is a double strand break repair protein that functions with or in parallel with BRCA1 in order to localize BRIP1 to sites of DNA breaks [24, 31]. MRE11 and its associated nuclease activity are necessary for efficient BRIP1 recruitment to laser-induced double strand break [24, 31].

The *BRIP1* gene is mutated in patients with Fanconi anemia (FA), a progressive bone marrow failure disorder, which is an autosomal recessive disease associated with an abnormal response to DNA damage [24, 32]. The BRIP1 protein operates in the FA pathway of interstrand cross-link repair and contributes to homologous recombination [24]. In patients with FA, it has been calculated that the estimated cumulative probability of development of a solid tumor was 76% by the age of 45 years [32]. Importantly, in these patients, the median age for cancer was 16, compared with 68 in the general population [32]. Patients with FA, a *BRIP1*-related disease, are prone to liver tumors [33]. In a systematic review on FA patients the calculated prevalence rate of liver tumors was 3% [32], thus 10-fold higher when compared with patients with chronic HBV infection (0.28%) [34]. Altogether, these data support the leading role of BRIP1 in liver carcinogenesis.

In our study, among the three *BRIP1* variants associated with HCC risk, two (rs4986764 and rs4986765) are located in the vicinity of a MutLα interaction domain suggesting their potential implication through the BRIP1/MutLα pathway [27]. BRIP1 is physically linked to MutLα complex (a heterodimer of MLH1 and PMS2), one of the main players in DNA mismatch repair (MMR) [27, 35]. BRIP1 interacts with MLH1 through its helicase domain [35]. BRIP1-MutLα interaction, but not BRIP1-BRCA1 interaction, is essential for the establishment of an MLH1-dependent interstrand crosslink-induced repair function of BRIP1 [27, 35]. Interestingly, *BRIP1* rs4986764 (p.Ser919Pro) is located in the vicinity of the DNA helicase domain VI (Figure 2) suggesting a potential functional consequence of this missense variant on the alteration of BRIP1 signaling pathway. Immunohistochemistry-based approach helps identifying tumors exhibiting microsatellite instability when staining is negative for MLH1, PMS2, MSH2 and/or MSH6 antibodies [36]. In this setting, immunohistochemistry is recommended as a screening method for Lynch syndrome among patients with endometrial cancer [37]. Using immunohistochemistry assay, BRIP1 expression was observed to be reduced in cervical adenocarcinoma compared with normal cervix tissue and was correlated with unfavorable outcome including lymph node metastases [38]. No data were reported on HCC patients.

Three GWAS conducted in Asian populations looked for genetic variants potentially associated with HBV- and HCV-related HCC risk but failed to decipher the pathogenic pathways associated with HCC [20–22]. The first GWAS compared 355 chronic HBV carriers with HCC and 360 chronic HBV carriers without HCC, all of Chinese ancestry, through genotyping 440,794 SNPs and identified one intronic SNP (rs17401966) in *KIF1B* (kinesin family member 1B) on chromosome 1p36.22 that was associated with HBV-related HCC (joint OR, 0.61) [20]. The *KIF1B* gene encodes a motor protein that transports mitochondria and synaptic vesicle precursors and mutations in this gene have been associated with Charcot-Marie-Tooth disease [39]. In the second GWAS, 95 HBV-infected HCC patients and 97 HBV-infected patients without HCC were compared using the Illumina Human610-Quad BeadChip [22]. Four SNPs (rs12682266, rs7821974, rs2275959, rs1573266) at chromosome 8p12 were associated with HCC risk with ORs ranging from 1.31 to 1.39 [22]. These four variants are not within a gene or within its vicinity. To identify potential genetic susceptibility factors in association with HCV-induced HCC, 721 individuals with HCV-induced HCC and 2,890 HCV-negative controls of Japanese origin were compared in the third GWAS using 432,703 autosomal SNPs [21]. No variant was associated with HCC risk [21]. These three GWAS used a hypothesis-free tag-SNP approach and did not reveal any association with the *BRIP1* locus or its vicinity. Many reasons can be advanced to explain this fact. First, GWAS genotyping platforms are designed to exploit linkage disequilibrium patterns which are population specific [40]. For instance, many variants associated with breast cancer in European-ancestry women showed only a weak or no association with breast cancer in other ethnic groups [41]. This highlights the importance of transethnic genome-wide association studies across worldwide populations of ethnically diverse genetic ancestries [42]. Interestingly, in our study, the minor allele frequencies of the top variant in the replication study on Africans (rs4986765) differed between African (9%) and European controls (30%). We used data from the 1000 Genomes Phase 1 v3 project for calculating the Fixation index (*F*_ST_) between Europeans and Africans on the whole set of 714,058 variants including the 37 variants on *BRIP1* [43, 44]. The median *F*_ST_ index between European and African subpopulations was 0.004 and the *F*_ST_ index for rs4986765 was 0.2196, which was highly suggestive of directional selection (upper outlier threshold, 0.0466) (Supplemental Table S4). Consistently, in the Exome Aggregation Consortium (ExAC; http://exac.broadinstitute.org/) database on 60,706 unrelated individuals, the ‘*A*’ allele of rs4986765 had a frequency of 33.52% in Europeans (Non-Finnish) versus only 9.52% in Africans. Hence, the data from ExAC support our findings in control populations from both derivation and validation studies. Second, the genomic coverage of hypothesis-free tag-SNP approach used in GWAS genotyping arrays do not cover all common variants, and thus leading to false-negative results [45]. At the opposite, the custom array used in our study intentionally focused on DNA repair genes, thus increasing the a priori probability of identifying genetic variants associated with risk of HCC on DNA repair genes.

Our study confirms the pathogenic cleavage between HCC related to chronic viral hepatitis and those occurring in patients with alcoholic cirrhosis [46]. A recent meta-analysis of individual participant data revealed that the genetic association between a missense variant of *PNPLA3* (rs738409) and HCC risk was more pronounced in patients with alcoholic liver disease in comparison with patients with chronic HCV infection [46]. In our study, *BRIP1* variants were associated with HCC risk in patients with viral cirrhosis but not among those with alcoholic cirrhosis. Furthermore, the validation study on African populations —with a less dominant etiology of alcoholic cirrhosis— found the same results.

Our study has several strengths. First, we performed a comprehensive assessment of DNA repair genes variants through a custom-array approach based on exhaustive literature and mechanistic reviews. Second, we reported the first association study on genetic variants associated with HCC risk in European-ancestry populations. Third, we demonstrated the potential implication of the *BRIP1* gene in HCV- and HBV-induced liver carcinogenesis in both European- and African-ancestry populations.

We need to acknowledge limitations. First, the GoldenGate Assay applies stringent requirements to the quality of genetic variants and may generate missing data. It is the reason why we applied stringent quality control procedure by removing samples and SNPs with call rates under 90%. It should be noted that genotypes determined with the Illumina^®^ GoldenGate^®^ platform were highly concordant with those determined with the standard Sanger sequencing approach for all the three *BRIP1* variants found in association with HCC, in our study (data not shown). Second, among patients with viral cirrhosis, those with HCC were 6 years older than controls. This age difference between cases and controls is inherent to case-control design of the study. Indeed, HCC is unusual before the age of 40 years and reaches its peak prevalence at the age of 70 years [2]. Nevertheless, *BRIP1* variants were independently associated with HCC risk even after adjusting for age and cirrhosis duration. Third, we have not performed functional studies for evaluating the influence of *BRIP1* variants on the inactivation of the FA pathway in HCC; however, in consistence with our results, a previous report showed genetic inactivation of the FA pathway in HuH-7 HCC cell lines [47]. Fourth, cirrhotic patients without HCC could be still at risk for developing HCC after the end of our study and this may lead to a misclassification bias. In fact, *BRIP1* variants were associated with HCC risk even after adjusting for cirrhosis duration in logistic regression analysis and also in Cox's time-dependent proportional hazards regression analysis. Furthermore, we have performed genetic association studies comparing HCC patients with non-cirrhotic HCC-free patients with HCV and/or HBV infection and confirmed the association between the *BRIP1* locus and HCC risk. Finally, *BRIP1* variants could have phenotype expression at the pathological or radiological level; however, these data were not fully available in our study to allow us studying genotype-phenotype correlations. Future studies should assess the influence of *BRIP1* variants at the clinical, pathological, and molecular levels.

Our study should deserve further interest for evaluating *BRIP1* genotype as a predictor of efficacy in forthcoming innovative therapeutics of HCC that will target pathways related to FA [48]. Among these drugs, Poly(adenosine diphosphate ribose) polymerase (PARP) inhibitors have been shown to be effective in cancer patients with mutations in homologous recombination repair genes, including *BRIP1* [49, 50]. In vitro PARP inhibition using ABT-888 (veliparib), an orally bioavailable PARP inhibitor, was directly effective on HCC cell lines and enhanced their radiosensitivity [51]. A phase 2 trial found an 88% response rate to olaparib, another PARP inhibitor, in patients with castration-resistant prostate cancers showing defects in DNA-repair genes, including FA pathway genes [52]. A major finding of this trial was that the optimal response to olaparib was observed in patients whose tumors had monoallelic *ATM* aberrations with mutations affecting the kinase catalytic domain [52]. Nevertheless, BRIP1 role as a key player in carcinogenesis and as a therapeutic target remains to be specifically assessed in HCC.

In conclusion, our results provide evidence for the potential implication of *BRIP1*, a master gene of DNA mismatch repair, on HCC risk in both European- and African-ancestry populations. It sheds new light on the genetic architecture of liver carcinogenesis in the setting of chronic viral hepatitis. Our results suggest investigating in experimental models the BRIP1 phosphorylation pathway and its interaction with BLM as a possible mechanisms of liver carcinogenesis. Previous preclinical studies have showed that PARP inhibition is a potentially promising therapeutic strategy for HCC. Our data suggest that the *BRIP1* genotyping should be used to predict the efficacy of forthcoming trials of HCC treatments that will target this pathway.

## MATERIALS AND METHODS

### Genetic variant selection for the ‘DNA repair genes’ custom array

Based on an exhaustive review of the literature, a total of 94 genes involved in DNA repair and genomic stability were included in the ‘DNA repair genes’ custom array (Supplemental Table S5). Selection criteria, validation process, and detailed array design are available in Supplementary Methods (See supplementary appendix).

### Study design and primary aim

We performed four case-control studies on 2,006 European- (Derivation#1 and #2 studies) and African-ancestry (Validation#1 and #2 studies) patients originating from several cohorts [CiRCE study (n=496), ANRS Genoscan study (n=398), Swiss Hepatitis C Cohort Study (n=572), Jean Verdier Hospital cohort (n=235), Benin-Togo cohort (n=305)] using an in-house designed array encompassing 94 genes on eight DNA repair pathways. The full description of the four case-control studies is reported in Supplementary Methods (See supplementary appendix). In both Derivation #1 and Validation #1 studies, HCC patients were compared with HCC-free cirrhotic patients. In both Derivation #2 and Validation #2 studies HCC patients were compared with HCC-free cirrhosis-free patients with HCV- or HBV-related liver disease. An overview of the study design is reported in Figure 1. The aim of the study was to look for potential genetic determinants associated with HCC risk through a fine mapping of genetic variants on DNA repair genes among patients with alcohol- or viral-induced liver disease.

### Genotyping and quality controls

Genomic DNA was extracted from frozen white blood cells using the Nucleon BACC3 kit (GE Healthcare, France) according to the manufacturer's instructions. Extracted DNA was dissolved in 200 μL Tris-HCl buffer (10 mmol/L, pH 8.0) containing 1 mmol/L EDTA and stored at −20°C until use. DNA concentrations were measured by using PicoGreen dSDNA quantification kit (Invitrogen, Carlsbad, CA, USA). Genotyping of all samples was performed with a total of 250 ng of genomic DNA using the Illumina BeadArray platform and GoldenGate Assay (Illumina Inc., San Diego, CA, USA) following the manufacturer's protocol [53]. This system uses a high-density BeadArray technology in combination with an allele-specific oligonucleotide extension, adapter ligation and amplification assay protocol. Briefly, biotinylated DNA was immobilized on paramagnetic beads and the pooled SNP-specific oligonucleotides in the OPA were annealed on the DNA. This hybridization step is followed by an extension and ligation step, connecting one of the allele-specific oligo with the locus-specific oligo to generate DNA templates which were amplified using universal fluorescently-labelled primers. PCR products are annealed to an array composed of beads carrying oligonucleotides complementary to the IllumiCode sequences in the locus-specific oligos that are used for recognition of each SNP site. The fluorescence of an array matrix carrying Cy3- and Cy5- labeled beads were generated with the two-channel scanner. Hybridization data intensity processing, clustering and genotype calling were performed using the genotyping module in the BeadStudio 2 software (Illumina, USA). We followed the STREGA recommendations (STrengthening the REporting of Genetic Association Studies, http://www.med.uottawa.ca/public-health-genomics/web/eng/strega.html) and used the GRCh37 Genome Reference Consortium Human genome build 37 as the reference assembles of human genome for mapping variants position. In the validation study, *BRIP1* variants were genotyped using high-resolution melting analysis (See Supplemental Methods in the supplementary appendix).

Genotyping quality controls were performed before statistical analysis. All genetic variants with a call rate <90%, a minor allele frequency <1%, and those with a deviation from Hardy-Weinberg equilibrium (Fishers’ exact *P*-value <10^−4^) were removed before the analysis. All participants included in the study had sample call rate >90%. We assessed duplicated samples and those showing cryptic relatedness by calculating identity-by-descent (IBD). By estimating the probability of sharing zero, one, or two alleles for any two individuals as P(Z=0), P(Z=1), and P(Z=2), respectively, a proportion of IBD was calculated using the following formula: PI-HAT = PI = P(Z=1)/2 + P(Z=2). PI-HAT value >0.99 was considered to be indicative of duplicate samples and PI-HAT threshold >0.2 was used to suggest cryptic relatedness. No sample was removed after IBD quality control. In order to identify ancestry outliers and population stratification, PCA on genomic data was performed on the study samples merged with 1kG project populations (AFR, AMR, ASN, and EUR) as reference populations [54]. Eigenvalues were calculated assuming an additive genetic model. Inspecting the first ten principal components, we identified 5 population outliers from the initial study and who have been excluded from subsequent analyses (See Supplemental Figure S2 in the supplementary appendix).

### Statistical analysis

All quantitative variables are described as medians and percentiles [Interquartile range (IQR), 25–75^th^ percentile]. All proportions are expressed as percentages with 95% confidence intervals (95% CI). The comparison of continuous variables between subgroups was performed using the Mann-Whitney *U* test. Due to the relatively low sample size in the Validation #1 study we performed haplotype association tests approach using a moving window with a dynamic width of 10 kb. The association was calculated on a ‘per block’ basis using the Chi-squared test and multiple testing correction was carried out using single value permutations approach with 10.000 permutations. We estimated the haplotype frequencies using the expectation/maximization (EM) algorithm with a maximum EM iterations of 50 and an EM convergence tolerance of 0.0001 [55]. In post-hoc exploratory analysis, haplotype trend regression was carried out in order to estimate the influence of HCV genotype on the association between genetic variants and HCC phenotype in a subset of patients with available data from the Derivation#2 study. Genetic variants located in the top significant haplotype block were tested for their association with HCC risk in subsequent analyses. The comparison of genetic variant frequencies between patients with HCC and non-HCC subjects was carried out using the Fisher's exact test for the allelic model with false discovery rate (FDR) for multiple testing correction since this method has been shown to be more powerful than the Bonferroni method, maintaining a false positive rate close to the nominal level [56]. Linkage disequilibrium pairwise analysis was performed using a matrix output for both the expectation-maximization (EM) algorithm and composite haplotype method (CHM) and D’ value [57]. Significant genetic variants were tested for their association with HCC risk in multivariate analysis using logistic regression and Cox proportional-hazards regression. Results were expressed as odds ratios (ORs) and hazard ratios (HRs) with their 95% CI, respectively. Multivariate analyses were performed using MedCalc for Windows, version 16.1 (MedCalc Software, Ostend, Belgium) on the basis of a two-sided type I error with an alpha level of 0.05. All quality control and genotypic analyses were performed using SNP & Variation Suite (SVS) 8.4.3 (Golden Helix, Inc. Bozeman, MT, USA).

†**The CiRCE Study Group includes**: (1) *CiRCE Coordination France*: J.P. Bronowicki, V. Di Martino, M. Doffoël, P. Hillon (Study coordinator), and G. Thieffin; *CiRCE Coordination China*: H. Wen, F.P. He, and X.M. Lu, P. Hillon and D. Vuitton; (2) *CiRCE Scientific Board*: J. Faivre (President), J.P. Cercueil, V. Cottet, D. Delmas, P. Ducoroy, L. Duvillard, M. Guenneugues, J.L. Guéant, F. Habersetzer, N. Latruffe, M. Manfait, P. Oudet, and G. Sockalingum; (3) *CiRCE pharmacologists*: P. Trechot, M.B. Valnet-Rabier, T. Trenque, and M. Tebacher-Alt.

**List of collaborators form the Jean Verdier Study Group and their affiliations**: Erwan Guyot^1,2^, Angela Sutton^1,2^, Valérie Bourcier^3^, Véronique Grando^3^, Marianne Ziol^4,5^, Nathalie Charnaux^1,2^, Michel Beaugrand^3,6^, Pierre Nahon^3,6^, and Nathalie Ganne-Carrié^3,6^.

^1^University Paris 13-UFR SMBH/INSERM, U1148, Bobigny, France; ^2^Biochemistry Unit, Jean Verdier Hospital, APHP, University Paris 13, Bondy, France; ^3^Liver Unit, Jean Verdier Hospital, APHP, University Paris 13, Bondy, France; ^4^Liver Biobank “CRB des hôpitaux universitaires PSSD”, Jean Verdier Hospital, APHP, University Paris 13, Bondy, France; ^5^Anatomic Pathology Unit, Jean Verdier Hospital, APHP, University Paris 13, Bondy, France; ^6^University Paris 13-UFR SMBH/INSERM, U1162, Génomique fonctionnelle des Tumeurs solides, Paris, France.

**List of collaborators for the French ANRS HC EP 26 Genoscan Study Group**: Bertrand Nalpas (Paris), Laurent Abel (Paris), Mona Monteanu (Paris), Laurence Bousquet (Paris), Yen Ngo (Paris), Pascal Lebray (Paris), Joseph Moussalli (Paris), Yves Benhamou (Paris), Dominique Thabut (Paris), Anaïs Vallet-Pichard (Paris), Hélène Fontaine (Paris), Vincent Mallet (Paris), Philippe Sogni (Paris), Jean-Baptiste Trabut (Paris), Marc Bourlière (Marseilles), Ioannis Theodorou (Paris), Jean-François Delfraissy (Paris), Thierry Poynard (Paris), Stanislas Pol (Paris).

**Members of the Swiss Hepatitis C Cohort Study Group**: Francesco Negro, Laurent Kaiser (Geneva), Darius Moradpour, Vincent Aubert (Lausanne), Jean-François Dufour, Fabio Giudici, Olivia Keiser, Meri Gorgievski (Berne), Markus Heim, Hans Hirsch, Marielle Rutquist, Pascal Benkert, Thomas Fabbro (Basel), Andreas Cerny (Lugano), Gladys Martinetti-Lucchini (Bellinzona), Raffaele Malinverni (Neuchâtel), Hans H. Siegrist (La Chaux-de-Fonds), David Semela, Pietro Vernazza, Patrick Schmid, Günter Dollenmaier (St. Gallen), Beat Müllhaupt, Philip Bruggmann, Elisabeth Probst-Müller (Zürich).

## SUPPLEMENTARY MATERIALS FIGURES AND TABLES


