# Supplementary material for: BRIP1 coding variants are associated with a high risk of hepatocellular carcinoma occurrence in patients with HCV- or HBV-related liver disease
Source: Oncotarget. 2016 Aug 17;8(38):62842–57. doi: 10.18632/oncotarget.11327 (PMC5609885; doi:10.18632/oncotarget.11327)
Supplement: Supplementary file 1 [file oncotarget-08-62842-s001.pdf]

BRIP1 coding variants are associated with a high risk of hepatocellular carcinoma occurrence in patients with HCV- or HBV-related liver disease

SUPPLEMENTARY DATA

Description of derivation (Derivation #1 and #2) and validation studies (Validation #1 and #2)

Derivation #1 study

This investigation is an ancillary study of the CiRCE multicenter case-control study, which aimed to assess the influence of metabolic, genetic, drugs, and environmental factors (alcohol, tobacco, viruses, and diet) on the risk of HCC among cirrhotic patients (1, 2). Since November 1st, 2008, cirrhotic European-ancestry patients with or without HCC were included in six university hospitals belonging to the Cancéropôle Grand-Est (CGE) consortium (Besançon, Dijon, Metz, Nancy, Reims, and Strasbourg). All participants gave their written informed consent and the protocol of the study was approved by the ethic committee of the University Hospital of Dijon (University of Burgundy, Dijon, France) who coordinated the CiRCE study research protocol at the national level (French National Research Agency, ANR-11-LABX-0021) (1, 2). Each study center followed the same study protocol (1, 2). Cases were defined as cirrhotic patients presenting with HCC. The diagnosis of cirrhosis was based on liver biopsy or, in the absence of liver biopsy, on typical clinical, morphological and biological data at the discretion of the physician according to international guidelines. HCC diagnosis was based on the European Association for the Study of the Liver (EASL) criteria (3). The absence of HCC in patients with liver cirrhosis at inclusion was assessed through high-quality imaging examinations (abdominal ultrasonography, abdominal computed tomography scan, or abdominal magnetic resonance imaging) and alpha-fetoprotein <100 ng/ml within the 2 months prior to inclusion (2). The diagnosis of alcoholic cirrhosis was based on an alcohol consumption >14 units/week for women and >28 units/week for men or a report of current or previous alcohol abuse (2). Exclusion criteria were as follows: patients under 35 years old in order to avoid the inclusion of patients whose cancers have genetic basis since this specific population is not representative of the large majority of patients thus introducing an analytical bias; progressive extrahepatic cancer; human immunodeficiency virus infection; acute alcoholic hepatitis; major somatic or psychiatric illness not compatible with inclusion in the CiRCE study; or non-HCC primary liver cancer (1, 2). Age, sex, etiology of cirrhosis (HCV or HBV), alcohol, and tobacco consumption were obtained through standardized questionnaires. Blood samples collected from cirrhotic patients were processed at each study center. Aliquots of frozen white blood cells were shipped on dry ice to the INSERM unit U954 laboratory "NGERE – Nutrition, Genetics, and Environmental Risk Exposure" for DNA extraction and genomic analyses. An overview of the study design is reported in Figure 1A.

Derivation #2 study

In the Derivation #2 study the 56 HCC patients with viral cirrhosis from the Derivation #1 study were compared with 970 HCC-free and cirrhosis-free patients with chronic HCV infection. These patients were recruited from two cohorts of adult patients of European descent from France and Switzerland. As previously described, the French cohort (ANRS Genoscan study group, n=398) included patients from the hepatology units of several hospitals in Paris and Marseilles (4). The Swiss Hepatitis C Cohort Study (SCCS, n=572) is a multicenter study of HCV-infected patients enrolled at eight major Swiss hospitals and the affiliated local centers. The French and Swiss cohorts were genotyped for ~350,000 variants and ~1,000,000 variants, respectively, using Illumina HumanCNV370-Duo and Human1M-Duo beadchips (Illumina, San Diego, USA) (4). Genotype imputation was performed in the French cohort (for the rs4986764 variant), using the Swiss cohort as a template. Only genotyping data for the three top variants retrieved in the Derivation #1 study (*BRIP1* locus; rs4986763, rs4986764, rs4986765) were used in the Derivation #2 study.

Validation #1 study

Two case-control comparisons were performed in the validation study. The Validation #1 study included patients who were consecutively referred to the Jean Verdier Hospital Liver Unit (JVH cohort) for diagnosis and management of cirrhosis between January 1999 and December 2007 (5). In order to replicate our initial results in African populations, only patients with African ancestry were included in the genotyping study. In the Validation #1 study, 136 HCC patients with viral-related cirrhosis from the JVH cohort were compared with 99 HCC-free patients with HBV- or HCV-related cirrhosis from the JVH cohort. All participants gave their informed consent. DNA samples were prepared from frozen blood samples stored in the Liver Biobank "CRB des Hôpitaux Universitaires Paris-Seine-Saint-Denis" BB-0033-00027, and were shipped on dry ice to the INSERM unit U954 laboratory for genomic analyses.

Validation #2 study

In the Validation #2 study, 136 HCC patients with viral-related cirrhosis from the JVH cohort were compared with 305 HCC-free and cirrhosis-free patients with HBV- and/or HCV infection recruited in Benin and Togo (Benin-Togo cohort) (6-10). Institutional review board approval was obtained from the ethical committees of the University Hospital of Nancy (Vandoeuvre-lès-Nancy, France), the University of Benin (Cotonou, Benin), and the University of Lomé (Lomé, Togo) (6-10). Written informed consent was obtained from participants.

Genetic variant selection for the 'DNA repair genes' custom array

Based on an exhaustive review of the literature, a total of 94 genes involved in DNA repair and genomic stability were included in the 'DNA repair genes' custom array [("dna repair"[MeSH Terms] OR ("dna"[All Fields] AND "repair"[All Fields]) OR "dna repair"[All Fields]) AND ("genes"[MeSH Terms] OR "genes"[All Fields]) OR ("polymorphism, genetic"[MeSH Terms] OR ("polymorphism"[All Fields] AND "genetic"[All Fields]) OR "genetic polymorphism"[All Fields] OR ("polymorphism"[All Fields] AND "genetic"[All Fields]) OR "polymorphism, genetic"[All Fields]) OR ("polymorphism, single nucleotide"[MeSH Terms] OR ("polymorphism"[All Fields] AND "single"[All Fields] AND "nucleotide"[All Fields]) OR "single nucleotide polymorphism"[All Fields] OR ("polymorphism"[All Fields] AND "single"[All Fields] AND "nucleotide"[All Fields]) OR "polymorphism, single nucleotide"[All Fields]) OR ("genome-wide association study"[MeSH Terms] OR ("genome-wide"[All Fields] AND "association"[All Fields] AND "study"[All Fields]) OR "genome-wide association study"[All Fields] OR ("genome"[All Fields] AND "wide"[All Fields] AND "association"[All Fields] AND "study"[All Fields]) OR "genome wide association study"[All Fields]) OR (("genetic therapy"[MeSH Terms] OR ("genetic"[All Fields] AND "therapy"[All Fields]) OR "genetic therapy"[All Fields] OR "genetic"[All Fields]) AND variant[All Fields])) AND ("neoplasms"[MeSH Terms] OR "neoplasms"[All Fields])]. Genetic variants were selected from available databases (NCBI, http://www.ncbi.nlm.nih.gov/guide; HapMap, http://hapmap.ncbi.nlm.nih.gov; and Ensembl, http://www.ensembl.org/index.html) based on evidence for their relationship with several malignancies, including HCC. We included genetic variants encoding non-synonymous amino acid substitutions that were suggested to alter protein function or those located within the 5' or 3' untranslated region of the gene that could potentially alter mRNA stability. Additionally, we included intronic variants in some instances to allow good genes coverage. We conducted a preliminary *in silico* validation phase of all identified genetic variants in order to determine their suitability for genotyping with the GoldenGate assay using the Illumina Assay Design Tool (ADT) at http://www.Illumina.com. The assessment of each genetic variant was conducted based on two Illumina's in-house criteria: 1) the designability score: 1, highly designable; 0.5, moderately designable; or 0, low designability; 2) the 60-bp limitation rule (a genetic variant cannot be closer than 60-bp to another one on the oligonucleotide pool assay, OPA). Genetic variants with low designability score were discarded and the final selection of the 384 SNPs was sent to Illumina for the synthesis of the OPA (also referred to as GoldenGate genotyping assay). Full description of the 'DNA repair genes' custom array is summarized in the Supplemental Table S1 (See supplementary appendix).

The 'DNA repair genes' custom array includes eight DNA repair gene pathways: **Pathway #1: direct reversal of damage** (*MGMT*); **Pathway #2: base excision repair** (*APEX1, MBD1, MBD2, MBD4, NEIL3, NTHL1, MUTYH, MPG, OGG1, PARP1, PARP2, PARP3, SMUG1, TDG, UNG, XRCC1*); **Pathway #3: nucleotide excision repair** (*ATXN3, CCNH, CDK7, ERCC1, ERCC2, ERCC3, ERCC4, ERCC5, ERCC6, ERCC8, GTF2H1, GTF2H4, MNAT1, LIG1, XAB2, DDB2, MMS19L, RAD23B, RPA1, RRM1, SLK, XPC*); **Pathway #4: double-strand breaks repair pathway** (*BRCA1, BRCA2, BRIP1, DCLRE1A, EME1, FANCA, FANCE, FANCI, FANCM, GEN1, LIG4, MUS81, NBN, PRKDC, RAD18, RAD51, RAD52, XRCC2, XRCC3, XRCC4, XRCC5*); **Pathway #5: mismatch repair** (*CHAF1A*, *MLH1, MLH3, MSH2, MSH3, MSH4, MSH6, N4BP2, PMS1, PMS2*); **Pathway #6: DNA polymerases, nucleases and helicases** (*BLM, EXO1, PCNA, POLB, POLD1, POLE, POLH, POLI, POLL, POLN, POLQ, RECQL4, REV1, REV3L, WRN*); **Pathway #7: telomere maintenance** (*TEP1, TNKS1BP1*), and **Pathway #8: DNA damage recognition and response** (*ATM, ATR, CHEK1, CHEK2, MDC1, RAD17, TP53*).

Polymerase chain reaction and high-resolution melting analysis

Primers for polymerase chain reaction high-resolution melting analysis (HRM) analysis to genotype BRIP1 variants (rs4986765, rs4986764, and rs4986763) were designed with μDESIGN (https://www.dna.utah.edu/udesign/app.php). The sequences of primer set were 5′- ATCTATCTATTTTACACCTGAACTTT 3′ (forward) and 5′-CATTTTTTTCTTCATCTGT ATCTTCAGGAT -3′ (reverse) for rs4986763, 5′-CACTTGAAGTGACCTCTTTAAAGTACAGTAC -3′ (forward) and 5′- TGCTTCCAGTAAATAAGGT -3′ (reverse) for rs4986764, and 5′- CATTCAA CCTTTGAAAGTGCACTGG -3′ (forward) and 5′-

GATGCTTTTTGGAAAATTCAGCCAAGG -3′ (reverse) for rs4986765.

The assay was performed on a LightCycler® 480 (Roche Applied Science) under the following conditions: pre-incubation at 95 °C for 10 min each, followed by 45 cycles of 95 °C for 10 s (denaturation) and 60 °C for 15 s (annealing with ramp rate of 2.20 °C/s and 1 step per cycle down to 50 °C), and extension step of 72 °C 10 s. Acquisition of the fluorescence signal was performed during each extension step. Amplification was followed by HRM analysis of the real-time PCR products, consisting of a denaturation step at 95 °C for 60 s, a cooling step at 40 °C for 60 s, a short hold at 65 °C for 1 s and a continuous acquisition step (with 25 acquisitions per °C) from 65 °C to 95 °C. The final cooling step consisted of a 10 s hold at 40 °C. HRM curves were classified into two or three distinct groups. Samples with known genotypes were used as internal references to generate standard curves for the classification of the unknown samples.

REFERENCES

1. Limagne E, Cottet V, Cotte AK, Hamza S, Hillon P, Latruffe N, et al. Potential role of oxidative DNA damage in the impact of PNPLA3 variant (rs 738409 C>G) in hepatocellular carcinoma risk. Hepatology. 2014; 60:1110-1.
2. Petit JM, Hamza S, Rollot F, Sigonney V, Crevisy E, Duvillard L, et al. Impact of liver disease severity and etiology on the occurrence of diabetes mellitus in patients with liver cirrhosis. Acta Diabetol. 2014; 51:455-60.
3. Bruix J, Sherman M. Management of hepatocellular carcinoma: an update. Hepatology. 2011; 53:1020-2.
4. Patin E, Kutalik Z, Guergnon J, Bibert S, Nalpas B, Jouanguy E, et al. Genome-wide association study identifies variants associated with progression of liver fibrosis from HCV infection. Gastroenterology. 2012; 143:1244-52 e1-12.
5. Tarhuni A, Guyot E, Rufat P, Sutton A, Bourcier V, Grando V, et al. Impact of cytokine gene variants on the prediction and prognosis of hepatocellular carcinoma in patients with cirrhosis. J Hepatol. 2014; 61:342-50.
6. Oussalah A, Besseau C, Chery C, Jeannesson E, Gueant-Rodriguez RM, Anello G, et al. Helicobacter pylori serologic status has no influence on the association between fucosyltransferase 2 polymorphism (FUT2 461 G->A) and vitamin B-12 in Europe and West Africa. Am J Clin Nutr. 2012; 95:514-21.
7. Bronowicki JP, Abdelmouttaleb I, Peyrin-Biroulet L, Venard V, Khiri H, Chabi N, et al. Methylenetetrahydrofolate reductase 677 T allele protects against persistent HBV infection in West Africa. J Hepatol. 2008; 48:532-9.
8. Gueant JL, Chabi NW, Gueant-Rodriguez RM, Mutchinick OM, Debard R, Payet C, et al. Environmental influence on the worldwide prevalence of a 776C->G variant in the transcobalamin gene (TCN2). J Med Genet. 2007; 44:363-7.
9. Gueant-Rodriguez RM, Gueant JL, Debard R, Thirion S, Hong LX, Bronowicki JP, et al. Prevalence of methylenetetrahydrofolate reductase 677T and 1298C alleles and folate status: a comparative study in Mexican, West African, and European populations. Am J Clin Nutr. 2006; 83:701-7.
10. Amouzou EK, Chabi NW, Adjalla CE, Rodriguez-Gueant RM, Feillet F, Villaume C, et al. High prevalence of hyperhomocysteinemia related to folate deficiency and the 677C-->T mutation of the gene encoding methylenetetrahydrofolate reductase in coastal West Africa. Am J Clin Nutr. 2004; 79:619-24.

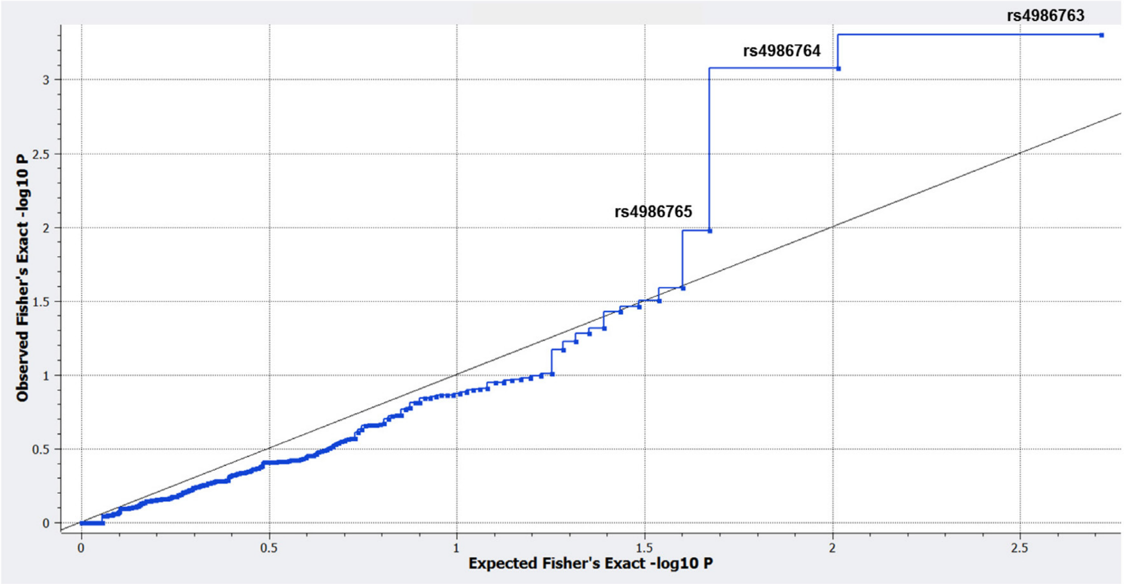

Supplementary Figure 1: Quantile-quantile (Q-Q) plot of the association results in the derivation #1 study (viral cirrhosis etiology).

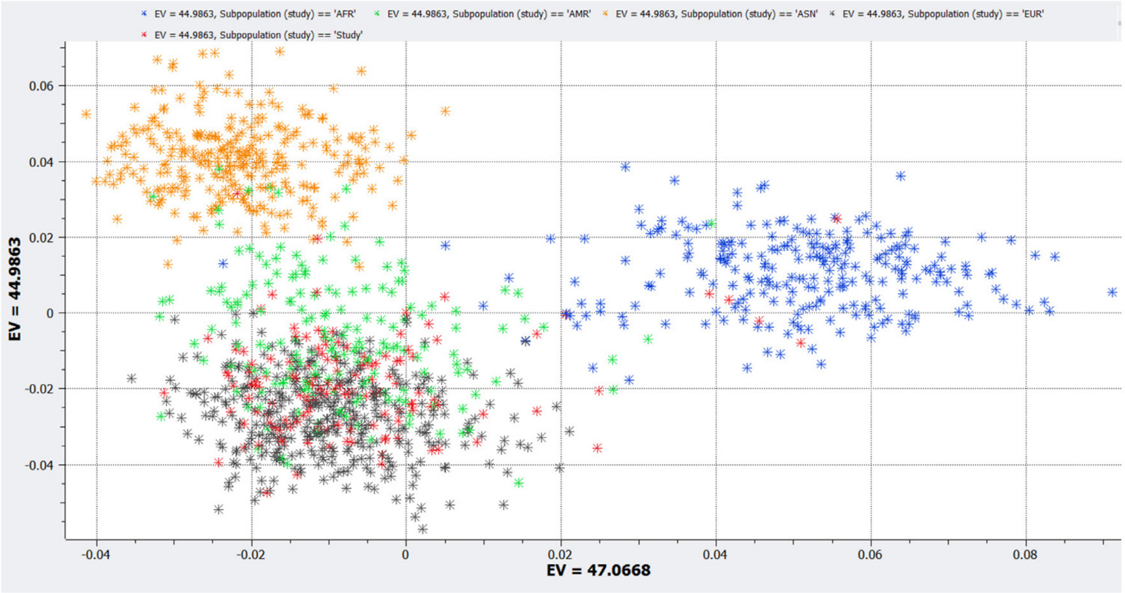

Supplementary Figure 2: Principal-component analysis (cases and controls). Principal-component analysis (PCA) was performed on the study samples merged with 1000 genomes populations as reference populations to identify ancestry outliers.

Supplementary Table 1: Baseline characteristics of alcoholic cirrhosis patients with and without hepatocellular carcinoma

See Supplementary File 1

Supplementary Table 2: Comparison of patients according to viral cirrhosis etiology in patients with viral-related liver disease from the Deviation #1 study

	HBV-related cirrhosis			HCV-related cirrhosis			
	n/N	%	95% CI	N	%	95% CI	P-value*
Male gender	20/23	87	72 to 100	80/115	70	61 to 78	0.147
HCC occurrence	13/23	57	35 to 78	42/115	37	28 to 46	0.120
	N	Median	25 - 75 P	N	Median	25 - 75 P	P-value†
Age (years)	22	62	53 to 71	115	63	52 to 72	0.765
BMI (kg/m²)	23	25.0	22.1 to 29.3	115	26.0	23.1 to 29.1	0.654
Waist circumference	20	98	89 to 110	96	96	87 to 104	0.355
ALAT	22	42	28 to 77	112	58	35 to 95	0.145
Platelets (G/L)	22	125	93 to 142	112	127	94 to 195	0.175
Creatinine (µmol/L)	23	84.0	70 to 94	112	82	69 to 95	0.682

NOTE. HBV: hepatitis B virus; HCV: hepatitis C virus; HCC: hepatocellular carcinoma; ALAT: alanine aminotransferase; BMI: Body mass index.
* Chi-square test.
† Mann-Whitney *U* test.

Supplementary Table 3: Array-wide haplotype association study for HCC risk in patients with viral-related liver disease from the Deviation #1 study

See Supplementary File 1

Supplementary Table 4: Fixation index (FST) between European and African subpopulations using data from the 1000 Genomes Phase 1 v3 project

See Supplementary File 1

Supplementary Table 5: Listing of the 384 genetic variants retained in the 'DNA repair genes' custom array (If the same variant falls in several transcripts within the same gene, a new row will be displayed for each transcript. Therefore, this number reflects the number of variant consequence types across the transcripts)

See Supplementary File 1
